# Hydrogen Bond Variations of Influenza A Viruses During Adaptation in Human

**DOI:** 10.1038/s41598-017-14533-3

**Published:** 2017-10-30

**Authors:** Jiejian Luo, Lizong Deng, Xiao Ding, Lijun Quan, Aiping Wu, Taijiao Jiang

**Affiliations:** 1 Key Laboratory of Protein & Peptide Pharmaceuticals, National Laboratory of Biomacromolecules, Institute of Biophysics, Chinese Academy of Sciences, Beijing, China; 2 University of the Chinese Academy of Sciences, Beijing, China; 3 Center for Systems Medicine, Institute of Basic Medical Sciences, Chinese Academy of Medical Sciences & Peking Union Medical College, Beijing, 100005 China; 4 Suzhou Institute of Systems Medicine, Suzhou, Jiangsu 215123 China

## Abstract

Many host specific mutations have been detected in influenza A viruses (IAVs). However, their effects on hydrogen bond (H-bond) variations have rarely been investigated. In this study, 60 host specific sites were identified in the internal proteins of avian and human IAVs, 27 of which contained mutations with effects on H-bonds. Besides, 30 group specific sites were detected in HA and NA. Twenty-six of 36 mutations existing at these group specific sites caused H-bond loss or formation in at least one subtype. The number of mutations in isolations of 2009 pandemic H1N1, human-infecting H5N1 and H7N9 varied. The combinations of mutations and H-bond changes in these three subtypes of IAVs were also different. In addition, the mutations in isolations of H5N1 distributed more scattered than those in 2009 pandemic H1N1 and H7N9. Eight wave specific mutations in isolations of the fifth H7N9 wave were also identified. Three of them, R140K in HA, Y170H in NA, and R340K in PB2, were capable of resulting in H-bond loss. As mentioned above, these host or group or wave specific H-bond variations provide us with a new field of vision for understanding the changes of structural features in the human adaptation of IAVs.

## Introduction

Influenza A viruses (IAVs) are negative-sense, single-stranded, and segmented RNA viruses, whose natural reservoir is wild aquatic bird. Currently, H1N1 and H3N2 IAVs co-circulate amongst human worldwide seasonally, which cause more than 5 million cases of severe illness and about 500 thousand deaths every year^1^. In theory, avian IAVs are not capable of infecting human because of the host-range restriction^2^. However, the emergence of human infections with avian H5N1 and H7N9 IAVs in these years demonstrates a potential pandemic threat^3,4^. Unfortunately, it is still unclear how IAVs adapted in different hosts. Previous researches have found that the HA protein plays a crucial role in the host adaptation because it binds to sialic acid receptors of host cells and mediates membrane fusion and viral entry^2^. In general, the HA proteins from human-adapted IAVs tend to bind a2,6-linked sialic acid linkages while those from avian-adapted IAVs prefer a2,3-linked sialic acid linkages^5^. In addition, other viral proteins, such as polymerase subunits, have also been reported as a determinant of host range of IAVs^2,6^.

In the last decade, computational or experimental researches have been carried out to identify singular or combinatorial host specific signatures of IAVs^7–10^, some of which were likely to facilitate the host adaptation process. However, the analysis of mutations generally focused on amino acid changes instead of structural variations of proteins. The hydrogen bond (H-bond) is one of the most important noncovalent interactions in biology which plays a significant role in stabilizing the three-dimensional structures and molecular interactions^11^. Previous studies have identified several mutations with H-bond variations in the process of host adaptation of IAVs. Xu *et al*. showed that the dual mutations E190D and G225D at the HA receptor binding sites switched the receptor specificity from avian-type to human-type in 2009 pandemic H1N1 (pH1N1) because of the formation of H-bond interactions between the glycan and HA^12^. In addition, the mutation H110Y which is located at the trimer interface forms a H-bond with the 413N of the adjacent monomer in order to stabilize the trimeric HA protein of H5 subtype^13^. The NA of H5N1 and pH1N1 with H274Y mutation significantly weakened the binding affinity for the anti-viral drug oseltamivir, which resulted from the loss of H-bond interactions between the oseltamivir and two residues of NA (178W and 152R)^14^. Moreover, co-mutations V344M and I354L in the PB2 subunit of pH1N1 enhanced binding affinity by creating additional H-bond contacts between PB2 cap binding domain and the pre-mRNA cap analogue m7GTP^15^. However, these researches, as stated, were specific to a few influenza subtypes and only covered a few of proteins. Here, the H-bond variations of host specific and group specific sites in viral proteins were systematically evaluated. The combinations of mutations and H-bond changes at these sites significantly varied among pH1N1, human-infecting H5N1 and H7N9. In addition, the wave specific sites of the fifth H7N9 wave and their corresponding effects on H-bonds were also investigated.

## Results

### The H-bond variations of host specific sites in the eight internal proteins

We assessed the H-bond variations of viral internal proteins between avian and human IAVs. As shown in Table S3, a total of 36999 non-redundant internal protein sequences (M1: 1635, M2: 2184, NP: 4482, NS1: 4479, NS2:1939, PA: 7733, PB1: 6973, PB2: 7574) were included in avian dataset. For human dataset, it contained two seasonal subtypes H1N1 and H3N2. There were 2781 sequences (M1: 165, M2: 253, NP: 294, NS1: 441, NS2: 171, PA: 411, PB1: 512, PB2: 534) in H1N1 and 14457 sequences (M1: 537, M2: 919, NP: 1442, NS1: 2478, NS2: 534, PA: 2643, PB1: 2737, PB2: 3167) in H3N2.

**Figure 1:**
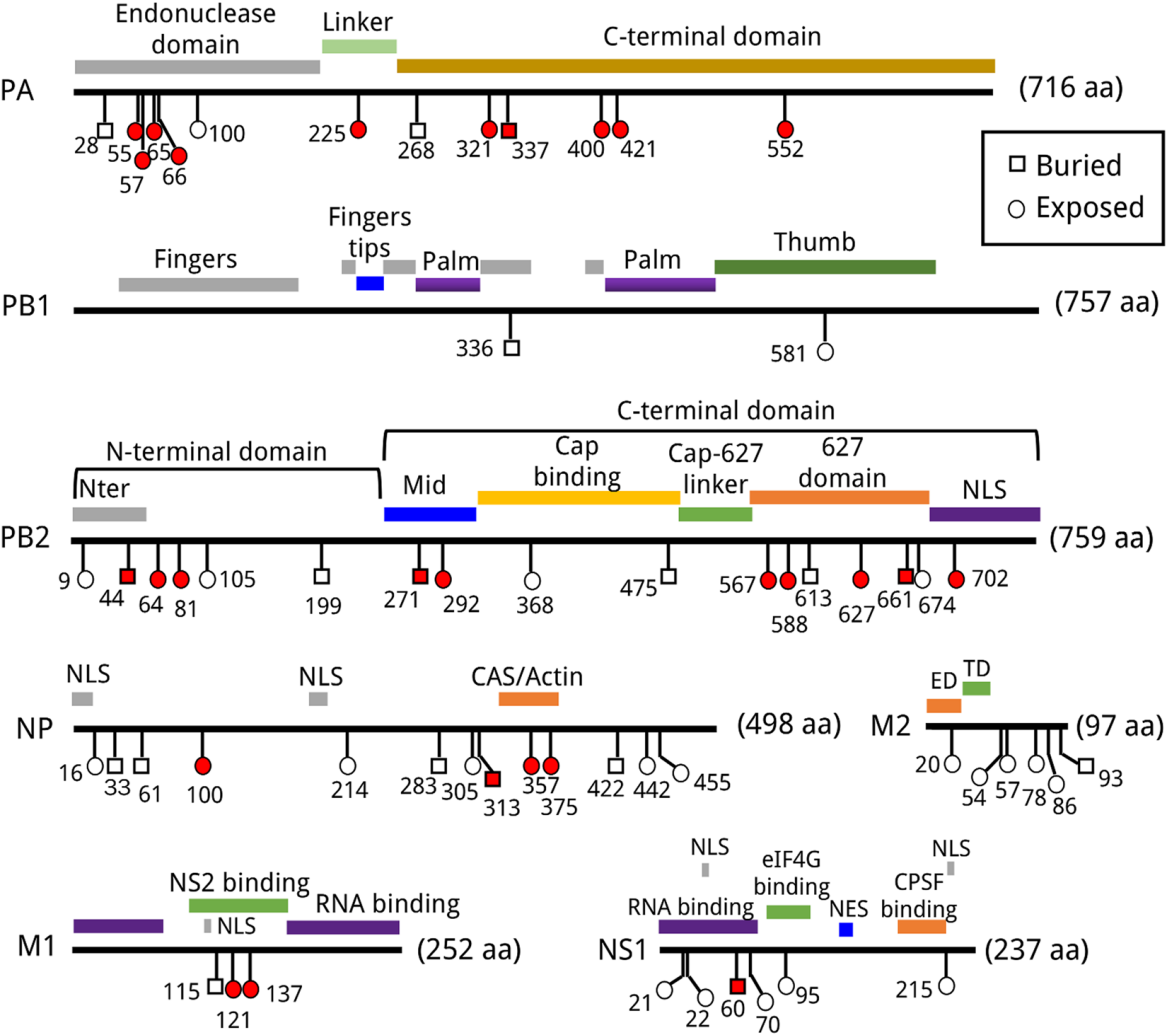
Linear mapping of host specific sites of internal proteins against known functional domains. Functional regions of proteins were highlighted with color bars. Buried sites and exposed sites were labeled as square and round, respectively. The sites with mutations leading to the H-bond loss or formation were colored as red. Nter: N-terminal. NLS: nuclear localization signal. NES: nuclear export signal. ED: extracellular domain. TD: transmembrane domain.

Sixty host specific sites were identified in the eight internal proteins (M1, M2, NS1, NS2, NP, PA, PB1, and PB2) of avian and human IAVs (Table 1). Over half of them (32/60) were in the viral RNA polymerase, including 13 sites in NP, 3 sites in M1, 6 sites in M2, and 6 sites in NS1. As host-associated positions reported in previous literatures^9,10^, the left two sites 70 and 107 of NS2 with dScore (0.89 and 0.88, respectively) below the threshold of 0.90 were excluded from our study. The H-bond variations of these host specific sites were evaluated through the differences of H-bond contacts with their neighboring residues between before and after a mutation. The relative solvent accessibility (RSA) of all the sites was calculated. Sites with RSA value above 25% were defined as exposed sites (located on the surface of protein). And then they were mapped onto the linear sequences of the proteins with functional annotations (Fig. 1). As shown in Fig. 1, 27 host specific sites contained H-bond loss or formation causing mutations including 10 sites in PA, 10 sites in PB2, 4 sites in NP, 2 sites in M1, and 1 site in NS1. There is no significant difference between the distributions of the H-bond variation sites and non H-bond variations sites on the three dimensional structures (the ratio of exposed sites: 77.8% in H-bond variation sites VS 63.6% in non H-bond variation sites, the two-tailed Fisher’s exact p-value is 0.27). As shown in Table 2, the number of sites at which mutations only gave rise to one kind of effect on H-bonds was 13 for H-bond loss and 10 for H-bond formation. The mutations at the other four sites could result in both H-bond loss and formation. For the convenience, all the H-bonds were written in the format of ‘HB(donor residue, acceptor residue, donor atom–H…acceptor atom)’. Notably, different mutations at the same site would lead to similar H-bond variations. Both S421I and S421V in the C-terminal domain of the PA protein could disrupted the H-bond HB(490R, 421S, Nη2–H…Oγ). Interestingly, 421I and 421V of PA were the dominant residue in H1N1 and H3N2, respectively. The same phenomena existed at NP 375, PA 65, and PB2 81 (Table 2).

**Table 1:** 60 host specific sites of internal proteins in avian and human IAVs.

Protein	Sites	Avian	Human		dScore	Protein	Sites	Avian	Human		dScore
			H1N1	H3N2					H1N1	H3N2	
M1	115	V	I	I	0.943	PA	28	P	L	L	0.974
	121	T	A	A	0.911		55	D	N	N	0.967
	137	T	A	A	0.929		57	R	Q	Q	0.952
M2	20	S	N	N	0.904		65	S	P (L)	L	0.975
	54	R	I (L)*	L	0.935		66	G	E	D	0.936
	57	Y	H (Y)	H	0.906		100	V	A	A	0.931
	78	Q	E (K)	K	0.955		225	S	C	C	0.955
	86	V	A	A	0.944		268	L	I	I	0.944
	93	N	S (N)	S	0.908		321	N	T (S)	Y	0.930
NP	16	G	D	D	0.954		337	A (T)	S	S	0.971
	33	V	I	I	0.920		400	P (SQ)	L	L	0.970
	61	I	L	L	0.954		421	S	I	V (I)	0.944
	100	R	V	V	0.990		552	T	S	S	0.971
	214	R	K	K	0.913	PB2	9	D	N	N	0.967
	283	L	P	P	0.954		44	A	S	S	0.962
	305	R	K	K	0.958		64	M (I)	T	T	0.961
	313	F	Y	Y	0.959		81	T	V (M)	M	0.965
	357	Q	K	K	0.989		105	T	V	V	0.971
	375	D	V (E)	G	0.928		199	A	S	S	0.981
	422	R	K (R)	K	0.918		271	T	A	A	0.983
	442	T	A (T)	A	0.911		292	I (V)	T	T	0.929
	455	D	E (D)	E	0.902		368	R (Q)	K	K	0.954
NS1	21	R	Q (R)	Q	0.926		475	L	M	M	0.974
	22	F	V	V	0.958		567	D	N	N	0.971
	60	A (E)	V	V	0.926		588	A	I	IT	0.908
	70	E (D)	K	K	0.943		613	V	T	A (T)	0.932
	95	L	V (I)	I	0.932		627	E	K	K	0.950
	215	P (S)	T	T	0.937		661	A	T	T	0.926
PB1	336	V	I	I	0.940		674	A	T	T	0.963
	581	E	D	D	0.926		702	K	R	R	0.928

*Minor amino acids with frequencies between 0.1 and 0.35 were shown in parentheses.

**Table 2:** H-bond variations at host specific sites of internal proteins.

Protein	Mutation	H-bond loss	H-bond formation
M1	T121A	HB(121T, 153Q, Oγ1-H...Oε1)*	
	T137A	HB(100Y, 137T, Oη-H...Oγ1); HB(137T, 134R, Oγ1-H...O); HB(138V, 137T, N-H...Oγ1)	
NP	R100V	HB(100R, 53E, Nη2-H...Oε2); HB(100R, 99R, Nη2-H...O)	
	F313Y		HB(313Y, 311Q, Oη-H...Oε1); HB(378T, 313Y, Oγ1-H...Oη)
	Q357K	HB(357Q, 484E, Nε2-H...Oε1); HB(357Q, 484E, Nε2-H...Oε2)	HB(357K, 484E, Nζ-H...Oε1); HB(357K, 484E, Nζ-H...Oε2)
	D375V/G/E	HB(376S, 375D, N-H...Oδ1)	
NS1	E60V	HB(10Q, 60E, Nε2-H...Oε1)	
PA	D55N		HB(55N, 59E, Nδ2-H...O)
	R57Q	HB(57R, 59E, Nη1-H...Oε1)	
	S65P/L	HB(65S, 67D, Oγ-H...Oδ1)	
	G66D		HB(51F, 66D, N-H...Oδ2)
	G66E		HB(52H, 66E, N-H...Oε2)
	S225C	HB(212R, 225S, Nη1-H...Oγ); HB(226L, 225S, N-H...Oγ); HB(227E, 225S, N-H...Oγ)	
	N321S/T	HB(321N, 319E, Nδ2-H...O)	
	N321Y	HB(321N, 319E, Nδ2-H...O)	HB(321Y, 319E, Oη-H...Oε1)
	A337S		HB(337S, 333N, Oγ-H...O)
	T337S	HB(337T, 333N, Oγ1-H...O)	HB(337S, 333N, Oγ-H...O)
	S400L	HB(400S, 272E, Oγ-H...O)	
	S421I/V	HB(490R, 421S, Nη2-H...Oγ)	
	T552S		HB(552S, 555G, Oγ-H...O); HB(553A, 552S, N-H...Oγ)
PB2	A44S		HB(44S, PA-580E, Oγ-H...Oε1); HB(44S, PB1-514V, Oγ-H...O)
	M/I64T		HB(64T, 61K, Oγ1-H...O); HB(64T, 65E, Oγ1-H...Oε2); HB(65E, 64T, N-H...Oγ1)
	T81V/M	HB(79S, 81T, Oγ-H...Oγ1)	
	T271A	HB(271T, 267V, Oγ1-H...O)	
	I/V292T		HB(292T, 291G, Oγ1-H...O)
	D567N	HB(569T, 567D, Oγ1-H...Oδ1)	HB(569T, 567N, Oγ1-H...Oδ1)
	A588T		HB(588T, 585P, Oγ1-H...O)
	E627K	HB(591Q, 627E, Nε2-H...Oε1)	
	A661T		HB(661T, PA-673R, Oγ1-H...O)
	K702R		HB(702R, 700E, Nη2-H...Oε2)

*The format of H-bonds is “HB(donor residue, acceptor residue, donor atom–H…acceptor atom)”.

### The H-bond variations of group specific sites for the HA and NA proteins

To analyze the H-bond variations in HA and NA, we also collected and selected HA and NA subtypes with more than 100 non-redundant sequences (Tables S1 and S4). For human dataset, 1560 H1, 10837 H3, 1393 N1, and 9512 N2 non-redundant protein sequences were included. For avian dataset, there were a total of 11284 HA proteins (H1: 139, H2: 295, H3: 819, H4: 820, H5: 3617, H6: 1080, H7: 1153, H8: 116, H9: 1951, H10: 489, H11: 498, H12: 159, H13: 148) and 9201 NA proteins (N1: 2321, N2: 2546, N3: 714, N4: 141, N5: 221, N6: 1239, N7: 459, N8: 1054, N9: 506).

**Figure 2:**
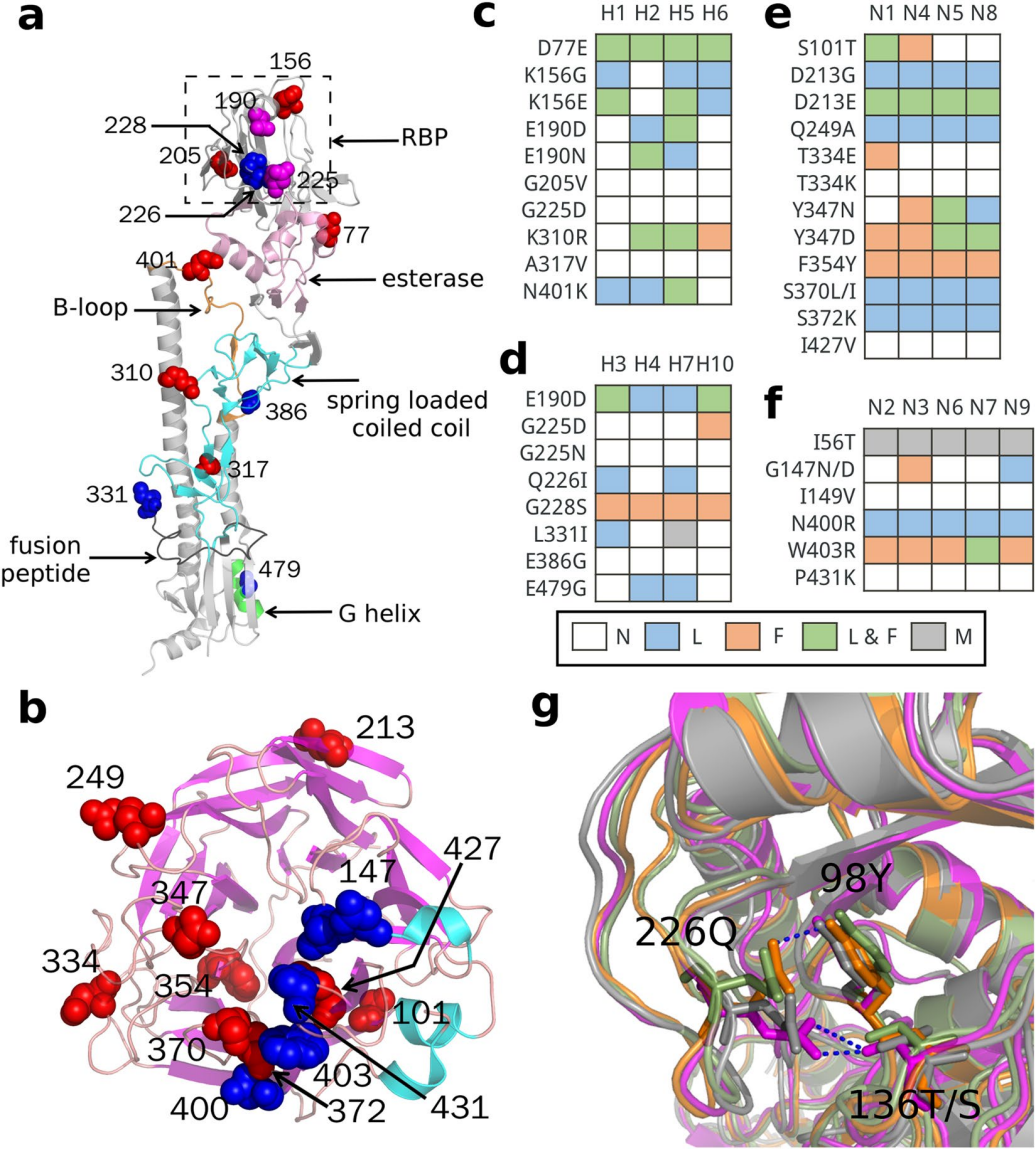
Group specific sites and their H-bond variations in HA and NA. (**a**) The distribution of group specific sites on the structures of HA (PDB:4O5N). Sites of group 1 HA and group 2 HA were colored as red and blue, respectively. The sites 190 and 225 in magenta at the receptor binding pocket (RBP) of HA were common in two groups. (**b**) The distribution of group specific sites on the structures of NA (PDB:3TIA). Sites of group 1 NA and group 2 NA were colored as red and blue, respectively. H-bond variations of group specific sites in subtypes of group 1 HA (**c**), group 2 HA (**d**), group 1 NA (**e**), and group 2 NA (**f**). The background colors of each cell were representative for the state of H-bond variations. white: No H-bond variations marked as N; light blue: H-bond loss marked as L; orange: H-bond formation marked as F; light green: both H-bond loss and formation marked as L & F; gray: the sites which were absent in predicted structures because of incomplete templates of crystal structures were marked as M. (**g**) Superposition model of RBP of H3 (orange), H4 (light green), H7 (magentas), and H10 (gray). Residues 226Q, 98Y, and 136T/S were shown in stick mode. HB(226Q, 98Y, Nε2-H…Oη) in H3, HB(136T, 226Q, Oγ1-H…Oε1) and HB(226Q, 137T, Nε2-H…Oγ1) in H7 were shown in blue dot line.

We were unable to detect any universal host specific site among all subtypes of the HA/NA protein. Then, the mutation analyses of the HA and NA proteins were done at the group level. Nevertheless, few sites could be identified when all the ten subtypes of the group 1 HA were considered (Table S1). To capture enough differential signatures in group 1 HA, we just selected H1, H2, H5, and H6 subtypes (a sub-group of group 1 HA) for calculation. The number of detected group specific sites detected in group 1 HA (H1, H2, H5, and H6 subtypes considered), group 2 HA (H3, H4, H7, and H10), group 1 NA (N1, N4, N5, and N8) and group 2 NA (N2, N3, N6, N7, and N9) was 8, 7, 9, and 6, respectively (Table 3). Notably, although sites 190 and 225 were overlapped in two groups of the HA protein, their amino acid usages in human infections were slightly different. For the HA protein of human infections (H1 lineage in group 1 and H3 lineage in group 2), the dominant residues of site 190 and 225 were both Asp. However, a certain proportion of human H1 possessed Asn at position 190 while some of human H3 had Asn at position 225 (Table 3). The H-bond variations related sites on protein structures between two groups for both HA and NA proteins were significantly different (Fig. 2a and b).

**Table 3:** Group specific sites of HA and NA.

Protein	Group^†^	Site^‡^	Avian	Human	dscore
HA					
	Group 1				
		77	D	E	0.957
		156	K	G (E)*	0.932
		190	E	D (N)	0.955
		205	G	V	0.987
		225	G	D	0.953
		310	K	R	0.955
		317	A	V	0.964
		401	N	K	0.978
	Group 2				
		190	E	D	0.983
		225	G	DN	0.916
		226	Q	I	0.972
		228	G	S	1.000
		331	L	I	0.992
		386	E	G	0.906
		479	E	G	0.960
NA					
	Group 1				
		101	S	T	0.929
		213	D	G (E)	0.987
		249	Q	A	0.983
		334	T	E (K)	0.960
		347	Y	N (D)	0.999
		354	F	Y	0.989
		370	S	L (I)	0.928
		372	S	K	0.988
		427	I	V	0.922
	Group 2				
		56	I	T	0.964
		147	G	N (D)	0.999
		149	I	V	0.976
		400	N	R	0.978
		403	W	R	0.992
		431	P	K	1.000

^†^Subtypes considered in groups. group 1 HA: Avian (H1, H2, H5, H6) and Human (H1); group 2 HA: Avian (H3, H4, H7, H10) and Human (H3); group 1 NA: Avian (N1, N4, N5, N8) and Human (N1); group 2 NA: Avian (N2, N3, N6, N7, N9) and Human (N2).

^‡^H3 numbering for HA and N2 numbering for NA.

*Minor amino acidSle with frequencies between 0.1 and 0.35 were shown in parentheses.

As shown in Fig. 2c–f, twenty-six of 36 mutations at the group specific sites caused H-bond loss or formation in at least one subtype, 17 of which didn’t share the same H-bond changes in all subtypes of the same group. For example, the mutation Q226I of group 2 HA led to H-bond loss in H3 and H7 subtypes, whereas it didn’t give rise to H-bond changes in H4 and H10 subtypes (Fig. 2d). In addition, the loss of H-bond contacts in H3 was HB(226Q, 98Y, Nε2-H…Oη) while those in H7 were HB(136T, 226Q, Oγ1-H…Oε1) and HB(226Q, 137T, Nε2-H…Oγ1) (Table S2). The residue at site 226 in the receptor binding pocket (RBP) of the HA protein was critical for receptor specificity for an avian or mammalian host^16^. We constructed the RBP superposition model and found that the local structures at position 226 in group 2 HA were different to some extend (Fig. 2g).

### H-bond variations in pandemic and sporadic human-infecting IAVs

**Figure 3:**
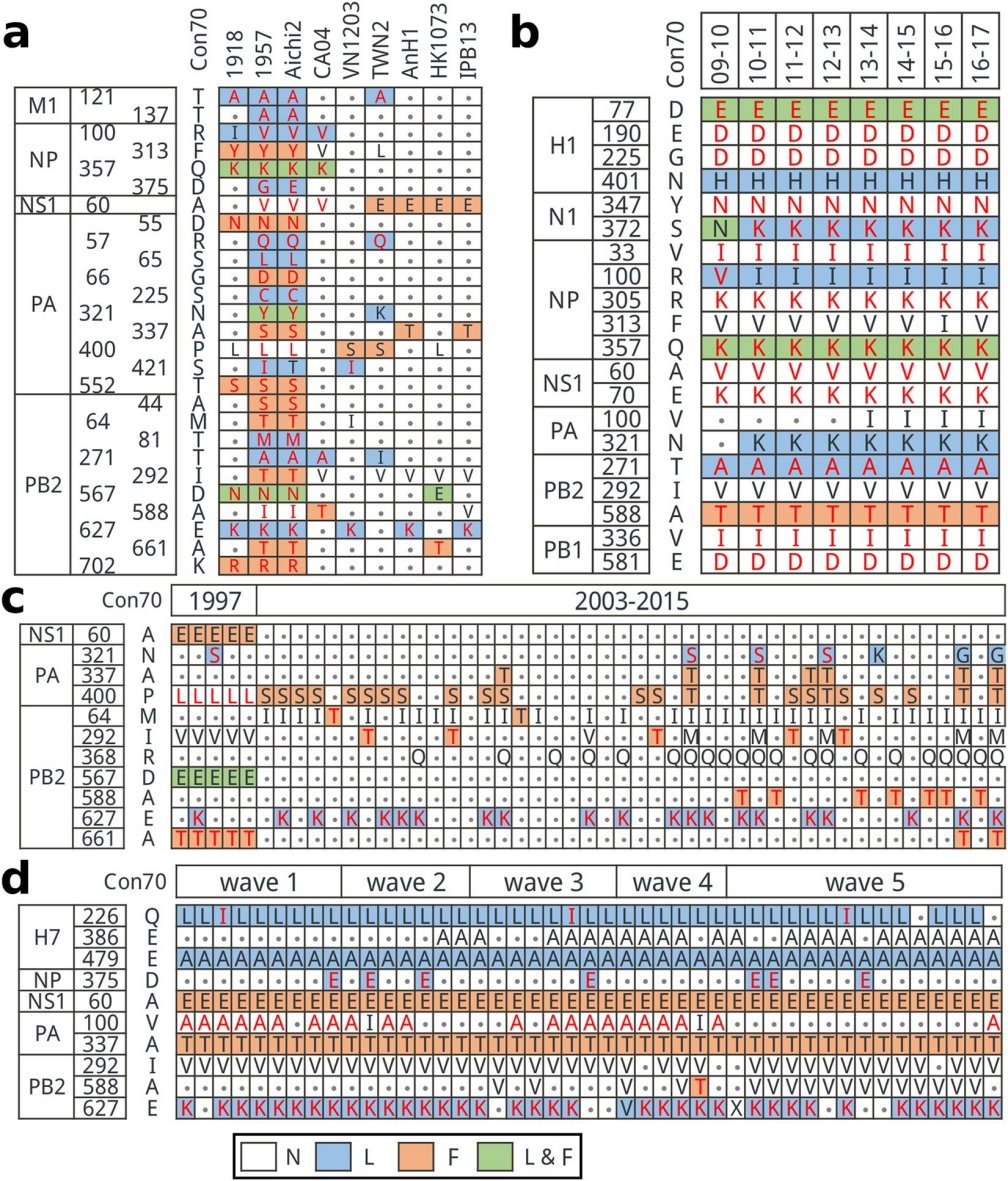
H-bond variations in pandemic and sporadic human-infecting IAVs. (**a**) The residue usages of representative isolations at the 27 H-bond variation sites. The representative strains were listed as follow. 1918: A/Brevig Mission/1/1918(H1N1); 1957: A/Albany/20/1957(H2N2); Aichi2: A/Aichi/2/1968(H3N2); CA04: A/California/04/2009(H1N1); VN1203: A/Viet Nam/1203/2004(H5N1); TWN2: A/Taiwan/2/2013(H6N1); AnH1: A/Anhui/1/2013(H7N9); HK1073: A/HongKong/1073/99(H9N2); IPB13: A/Jiangxi/IPB13/2013(H10N8). (**b**) Dominant mutations and their H-bond variations in pH1N1 from 09–10 season to 16–17 season. (**c**) Mutations and their H-bond variations in human-infecting H5N1 isolations collected from 1997 to 2015. (**d**) Mutations and their H-bond variations in the five waves of H7N9 from 2013 to 2017. The columns of (**c**) and (**d**) were representative strains arranged chronologically. The background color of each cell was representative for the state of H-bond variations. white: No H-bond variations marked as N; light blue: H-bond loss marked as L; orange: H-bond formation marked as F; light green: both H-bond loss and formation marked as L & F. Con70 means the consensus sequence (70% threshold) of avian IAVs. Residues matched with Con70 were represented with dots. Residues prefer existing in human H1N1 and H3N2 (Human-preferential residues) were in a red font. Representative strains in (**c**) and (**d**) were listed in Tables S6 and S7, respectively.

The comparison of the H-bond variations between pandemic and sporadic human-infecting IAVs was performed in Fig. 3a. Among the four pandemic representative isolations, A/Albany/20/1957 (H2N2, 1957) and A/Aichi/2/1968 (H3N2, Aichi2) contained more human-preferential mutations at the 27 H-bond variation sites than A/Brevig Mission/1/1918 (H1N1, 1918) and A/California/04/2009 (H1N1, CA04). For the five representative human-infecting IAVs, mutations were sporadic and their combinations were significantly different from the four pandemic strains.

The reassortant pH1N1 has co-circulated with H3N2 seasonally since 2009, but it is different from the seasonal H1N1 before 2009^17^. In the meantime, H5N1 and H7N9 IAVs are the two major subtypes of avian IAVs that can cause large-scale infections in human and poultry^18^. As reported in previous researches, the pattern of the spread of H5N1 in humans and birds around the world is consistent with the wild bird migration and poultry trade activities. In contrast, human cases of H7N9 and isolations of H7N9 in birds and the environment have largely occurred in a number of contiguous provinces in south-eastern China^18^. In addition, it has been found that the H7N9 cases are mainly among older cohorts while H5N1 cases are among younger cohorts^19^. Thus, it was necessary to do further comparisons of these three subtypes of IAVs to investigate their adaptations to human.

The number of the host specific or group specific sites with mutations in isolations of pH1N1, human-infecting H5N1 and H7N9 was 20, 11, and 10, respectively (Fig. 3b–d). Besides, the combination patterns of mutations and H-bond changes in these three subtypes of IAVs were also different. There were more mutations in the NP protein of pH1N1 than those of H5N1 and H7N9. As shown in Fig. 3b, there were 8 mutations that caused H-bond loss or formation in all seasons of pH1N1 except NA S372K and PA N321K. The amino acids at both NA 372 and PA 321 were Asn in isolations of season 09–10, which were replaced by Lys from season 10–11 on. It was obvious that the mutations of H5N1 distributed more scattered than those of pH1N1 and H7N9. The H5N1 infections in human were divided into two emergences. The first emergence was in Hong Kong in 1997 and the re-emergence was in Mainland China in 2003^18^. Two mutation patterns in H5N1 matched with these two emergences (Fig. 3c). There were five mutations with H-bond variations in most of the H7N9 isolations, among which HA Q226L/I, HA E479A, and PB2 E627K caused H-bond loss while NS1 A60E and PA A337T resulted in H-bond formation (Fig. 3d). The patterns of H-bond variations between H5N1 and H7N9 were significantly different (Fig. 3c and d). The NS1 A60E and PB2 E627K were the two common mutations in both H5N1 and H7N9 viruses. The NS1 A60E causing the H-bond formation was mainly existed in H5N1 strains collected in 1997 and H7N9 strains while H5N1 strains collected after 2003 preferred amino acid Ala at NS1 60. Less H5N1 isolations possessed the E627K mutation than the H7N9 strains. In addition, the other mutations that caused H-bond loss or formation in H5N1 were more sporadic than those in H7N9.

### Wave specific mutations and H-bond variations in the fifth H7N9 wave

**Figure 4:**
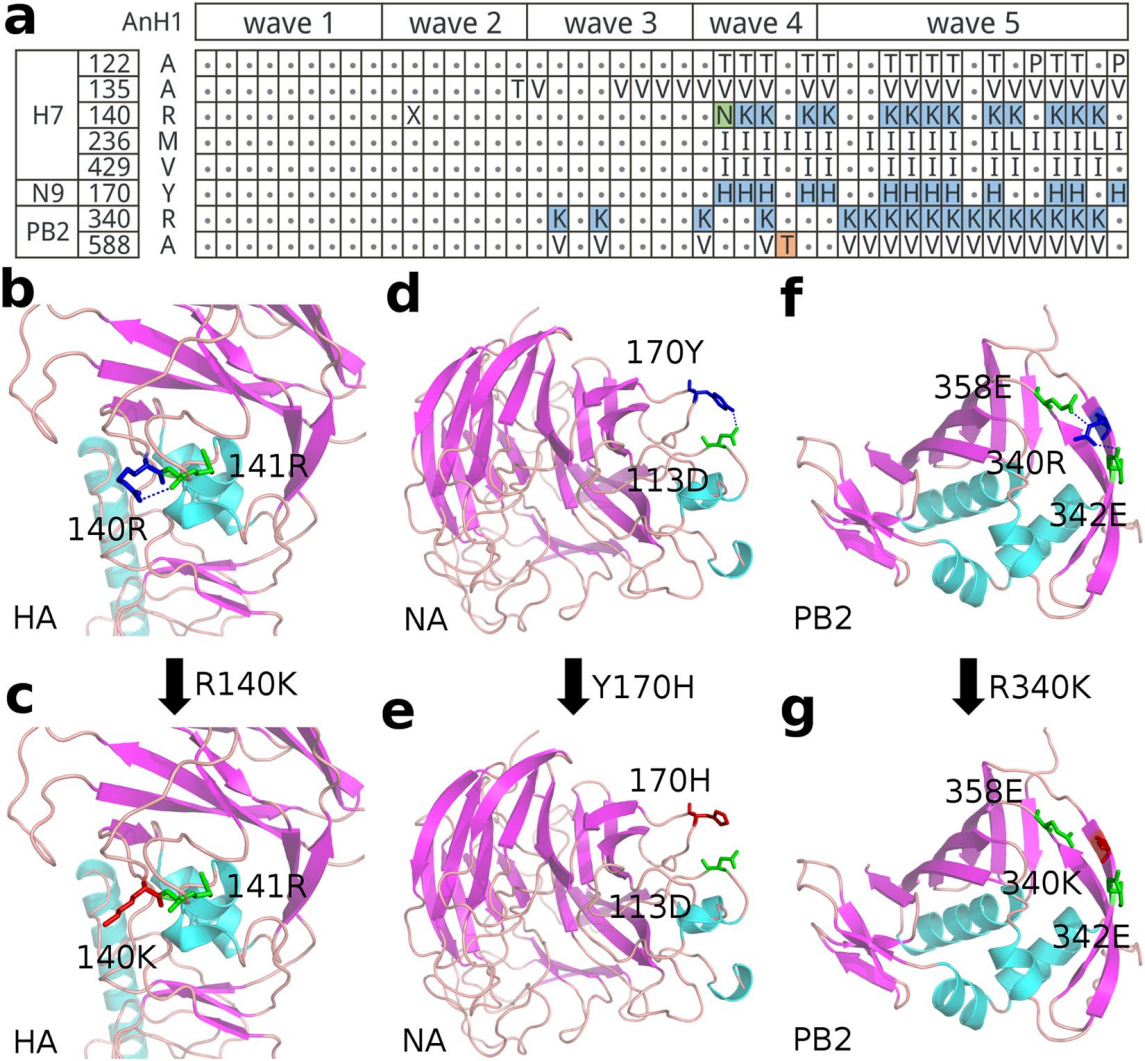
Wave specific mutations and H-bond variations in the fifth H7N9 wave. (**a**) Residue usages and H-bond variations at the eight wave specific sites of the fifth wave. Residues matched with the strain A/Anhui/1/2013 (H7N9, AnH1) were represented with dots. The background color of each cell was representative for the state of H-bond variations. white: No H-bond variations; light blue: H-bond loss; orange: H-bond formation; light green: both H-bond loss and formation. (**b** and **c**) Mutation R140K of HA and the loss of HB(140R, 141R, Nη1-H…O). (**d** and **e**) Mutation Y170H of NA and the loss of HB(170Y, 113D, Oη-H…Oδ2). (f and g) Mutation R340K in cap binding domain of PB2 and the loss of HB(340R, 358E, Nε-H…Oε2) and HB(340R, 342E, Nη2-H…Oε2). The helix, sheet and loop were colored by cyan, magentas and orange, respectively. H-bonds were shown in blue dot line. Representative strains in (**a**) were listed in Table S7.

The H7N9 virus has caused five epidemic waves of human infections in China since its first emergence in 2013. It was noted that elevated morbidity and mortality in a wider affected area were observed in the fifth wave^20^. To investigate the extraordinary phenomenon for the fifth wave, mutation comparison was also performed between the H7N9 isolations derived from wave 5 and from wave 1–3 (sequences data of wave 4 were not enough). As shown in Table 4, 8 mutations specific to the fifth wave were identified, which contained 5 mutations in the HA protein, 1 mutation in the NA protein and 2 mutations in the PB2 protein. The mutations R140K in the HA protein, Y170H in the NA protein and R340K in the PB2 protein were capable of causing the H-bond loss (Fig. 4a). The location of the residue 140R was in the 123–149 loop which was near the conserved B-cell epitopes 123–134 region (MGFTYSGIRTNG) of avian H7 HA. The mutation from Arg to Lys at position 140 disrupted the H-bond interaction HB(140R, 141R, Nη1-H…O) (Fig. 4b and c). The mutation Y170H in the NA protein caused the loss of a H-bond between the side-chains of Tyr-170 and Asp-113 which was in 163–172 loop (LSSPPTVYNS) and 111–120 loop (SSDVLVTREP) respectively (Fig. 4d and e). Besides, these two residues were both located on the interface of two subunits of NA (Figure S2). In addition, the R340K mutation in the PB2 protein could break the two H-bond interactions with its neighbor residues 358 and 342 (Fig. 4f and g). It is known that the Lys-340 in the cap bind pocket of PB2 played critical roles in mammalian adaptation of the H10N8 virus and viruses harboring PB2-588V exhibited higher polymerase activity^21^. As it happened, a majority of the H7N9 strains in the fifth wave contained both 340 K and 588 V in PB2 (Fig. 4a).

**Table 4:** Wave specific mutations in the fifth H7N9 wave.

Protein	Site	Wave 1-3 (n = 439)^†^	Wave 5 (n = 132)	Mutation
H7				
	122	A (99.5%)^‡^	T (75.0%); A (18.2%)	A → T
	135	A (80.9%); V (16.9%)	V (96.2%)	A → V
	140	R (95.2%)	K (87.9%); R (12.1%)	R → K
	236	M (96.8%)	I (84.8%); L (10.6%)	M → I/L
	429	V (100.0%)	I (86.4%); V (13.6%)	V → I
N9				
	170	Y (99.8%)	H (76.5%); Y (23.5%)	Y → H
PB2				
	340	R (92.0%); K (7.7%)	K (77.3%); R (22.7%)	R → K
	588	A (92.0%); V (7.7%)	V (76.5%); A (22.7%)	A → V

^†^The number of strains.

^‡^The ratio of a residue.

To investigate the difference between the Yangtze River Delta lineage (YRD) and the Pearl River Delta lineage (PRD), we collected the strains of H7N9 from wave 3 to wave 5 and constructed the approximately-maximum-likelihood phylogenetic tree of HA protein (Figure S6). The PRD and YRD lineages of the fifth H7N9 wave were colored as yellow and blue bar in Figure S6, respectively. Finally, 9 PRD and 114 YRD strains with completed genomes in the fifth H7N9 wave were selected.

As shown in Table S8, differential sites and H-bond variations between the PRD and YRD lineages in the fifth H7N9 wave were evaluated. The H-bond variations were assessed with the A/Anhui/1/2013 as reference. In total, 19 differential regions or sites were found, including 8 sites in HA, 1 site in M1, 1 site in M2, 8 sites in NA, and 1 site in PA. The insertion of basic amino acid residues RKRT at the cleavage site connecting the HA1 and HA2 peptide region was found in all the 9 PRD strains of wave 5, which was a signature of highly pathogenic avian influenza viruses^22^. In addition, the number of H-bond variations sites between PRD and YRD of wave 5 was 9, including 3 sites in HA, 5 sites in NA, and 1 site in PA (Table S8). For the lack of sufficient PRD sequences of wave 5 H7N9, the differences or signatures between PRD and YRD linages found here needed to be further validated.

## Discussion

Typical avian IAVs don’t have the capacity to replicate efficiently and cause human infections. In order to become capable of establishing in human, avian IAVs must overcome species barriers and adapt to a new host environment. Some changes need to be done to maintain the stability or function of viral proteins during the human adaptation of IAVs^2^. The H-bond is one of the most important noncovalent interactions for protein stabilization and molecular interactions^11^. Investigation of the changes of H-bond features will promote understanding the mechanism of viral adaptation in human.

In our analysis, 60 host specific sites of internal proteins between avian and human IAVs were identified, 27 of which contained mutations with effects on H-bonds. 75% (45/60) of the host specific sites were in the RNA polymerase and NP proteins. The RNA polymerase is responsible for the transcription and replication of the virus genome, while the NP encapsulates the virus genome to form a ribonucleoprotein (RNP) particle for the purposes of transcription and packaging^23^. It is well documented that polymerases from avian IAVs don’t function well in mammalian host^24^. This high proportion of mutations in the RNP might play important roles in viral adaptation in human. H3N2 and H1N1 are two major lineages of human IAVs. Despite their common origin, the internal protein sets of these two lineages have evolved independently^25^. Some sites with different residue usages in H1N1 and H3N2, such as PA 421 and NP 375, had the same effects on H-bonds (Table 2), which suggests the diversity of human adaptation. Group specific sites were further identified in HA and NA, which were shown in Table 3. The H-bond variations of some mutations at group specific sites of the HA/NA proteins were different among different subtypes (Fig. 2c–f). On the one hand, it might result from the differential local structures. On the other hand, these mutations had other important functions we haven’t yet discovered besides the H-bond contacts in the protein. Although the mutation Q226I was identified in H3, H4, H7, and H10 subtypes, the H-bond variation only emerged in the H3 and H7 subtypes. The local structural difference of 226Q was clear in the RBP superposition model of H3, H4, H7, and H10 subtypes (Fig. 2g). It has been well proved that Q226I/L in the RBP increased the ability to bind a2–6 sialic acid^26–30^, which implies its role in inter-molecular interactions is more important than that in intra-molecular interactions in HA. Besides, dual mutations E190D and G225D of receptor binding sites had no effect on H-bonds in H1 HA according to our assessment (Fig. 2c), but they mediated several H-bonds interactions between the glycan and HA to switch the receptor specificity from avian to human in H1N1 subtypes^12^. In other words, mutations with no effect on H-bonds in viral proteins might be an important part of inter-molecular interactions. Unfortunately, we were unable to evaluate these inter-molecular interactions systematically due to the insufficient structural information of protein or molecular interaction.

It is worth noting that we couldn’t give an evaluation of H-bond variations due to co-mutations or multiple mutations in this study. The mutation E627K in the PB2 627 nuclear localization signal (NLS) domain of the RNA polymerase disrupted the sidechain-sidechain H-bond interaction with 591Q (Table 2). Actually, it’s known that PB2 E627K can alter the surface electrostatic potentials of PB2-627NLS domain with the assistance of residues at 590 and 591^31^. According to our statistics, the major amino acids at 590 and 591 of PB2 in both avian and human IAVs were Gly and Gln, respectively. In the meantime, we assessed the effect of the mutation on H-bonds based on the avian consensus sequence environment without consideration of residue combinations in the real strains. Besides, the crystal structure of RNA polymerase of IAVs used in homology modeling was bat origin (A/little yellow-shouldered bat/Guatemala/060/2010, H17N10, PDB ID :4WSB) and its chain sequences were greatly different from the ones in other subtypes, which might result in structural differences. Therefore, more accurate and suitable crystal structures are needed to validate these H-bond variations resulting from adaptive mutations.

Both H5N1 and H7N9 have caused sporadic human cases without any evidence of sustained and human-to-human spread, but their patterns of H-bond variations were significantly different (Fig. 3c and d). In fact, the pattern of the spread of H5N1 in humans and birds around the world is consistent with the wild bird migration and poultry trade activities. In contrast, human cases of H7N9 and isolations of H7N9 in birds and the environment have largely occurred in a number of contiguous provinces in south-eastern China^18^. Besides, it has been found that the H7N9 cases are mainly among older cohorts while H5N1 cases are among younger cohorts, and the lifelong protection against H5N1 and H7N9 is via different childhood hemagglutinin imprinting^19^. So the different epidemic patterns of IAVs and different human immune responding to IAVs may be the possible explanations of differential patterns of mutations and H-bond variations between H5N1 and H7N9.

The H7N9 virus has caused five waves of human infections in China since March 2013. An increased pathogenicity in a wider affected area was observed in the fifth wave^20^. There is a sufficient preponderance of observed mutations in isolates of the fifth wave when compared with those in wave1–3 at the eight characteristic sites (Table 4). The mutations were acquired in the several strains of wave 4 (Fig. 4a), but we were not sure whether their frequencies were similar to those in wave 5 due to lack of enough sequences. The dual mutations, R340K and A588V in PB2, appeared in most of the isolations of wave 5 (Fig. 4a). The substitution from Arg to Lys at position 340 could disrupt the H-bond interactions with 358E and 342E in the cap binding domain of PB2 (Fig. 4f and g). In fact, both Arg and Lys were basic amino acids with similar chemical properties, whereas their differences of side-chain conformations at PB2 340 were clear. Because all the H-bond calculations were based on homology modeling using CISRR in our study, these H-bond variations need further validation in accurate crystal structures. The mutation from Lys to Asn reduced polymerase activity of A/Hamburg/NY1580/09 strain^32^. The residue 588V, located in the PB2 627-domain near the polymorphic 590 and 591 residues, had been reported that it is important for H7N9 and H10N8 virus replication and virulence^21^. The dual mutations R340K and A588V in PB2 might be a feature of the fifth wave of H7N9.

In summary, our study gave a systematic assessment of intra-molecular H-bond interactions at host specific or group specific sites between avian and human IAVs, which is helpful for us to understand human adaptation of IAVs from a new perspective. Of course, the H-bond interaction is just one kind of the noncovalent interactions. The effect of mutations may be multi-functional and they tend to function together. Therefore, more efforts need to be put into the study of the variations of structural features to get a comprehensive understanding of how these mutations work.

## Material and Methods

### Datasets

We retrieved all full-length sequences for ten proteins (HA, NA, NS1, NS2, M1, M2, NP, PA, PB1, and PB2) of IAVs isolations between 1918 and June 2017 from the GISAID database (http://platform.gisaid.org/epi3/frontend) and the Influenza Virus Database in NCBI^33^. Sequences from these two databases were merged. For human IAVs, we mainly considered epidemic seasonal H1N1, H2N2 and H3N2 strains. The human H2N2 contained less than 50 non-redundant sequences in each protein (Table S3) and this subtype was not considered in our statistical analysis. As reported in previous researches, the internal protein of human H1N1 and H3N2 have evolved independently^25^. It’s necessary to compare these two subtypes separately. Our investigation was focused on H-bond variations between avian and human IAVs and how these features changed after avian IAVs overcome host barriers to establish sustained infections in human. The effect of IAV reassortments should be excluded. Thus, the pandemic strains (1918 H1N1pdm, 1957–1958 H2N2pdm, 1968 H3N2pdm, and 2009 H1N1pdm) were removed from human dataset as they were reassortants^17,34–36^. For avian IAVs, all subtypes of IAVs were considered except suspicious subtype H1N1, H2N2, and H3N2 which also circulated amongst human. Besides, those strains annotated as mixed subtypes or lab strains were also excluded. Moreover, we did additional sequence cleaning for NS segment. NS gene could be grouped into two major variants known as allele A and B and human NS basically belonged to allele A^37,38^. So it was reasonable to remove allele B sequences before comparison of NS segment between avian and human. Sequence identities between allele A and B were about 70%, whereas those within each allele were above 90%^39^. We eliminated allele B sequences according to identities, in which case the NS of A/tern/South Africa/1961 (allele A; accession: CY014988) and A/redhead duck/ALB/74/1977 (allele B; accession: CY004739) were chosen as references. Reassortants at a given period such as 2009 pandemic H1N1 appear to be highly similar. We collapsed identical sequences for each protein with identity threshold equal to 1 using cd-hit^40^. This step is necessary and important for reduce the proportion of potential unknown reassortants in our dataset, although the frequency of IAV reassortments was low and few inter-subtype reassortants have actually established sustained infections in human^41,42^. Finally, we got a non-redundant dataset that comprised human IAVs (human-host H1N1 and H3N2) and avian IAVs (avian-host subtypes excluding H1N1, H2N2 and H3N2) (Table S3).

It is widely known that the HA protein can be divided into two groups: group 1 HA (H1, H2, H5, H6, H8, H9, H11, H12, H13, H16, H17, H18) and group 2 HA(H3, H4, H7, H10, H14, H15)^43^. The NA protein also has two groups: group 1 NA (N1, N4, N5, N8) and group 2 NA (N2, N3, N6, N7, N9)^44^. The two groups of HA can be further divided into several subgroups (Tables S1 and S4). The H14, H15, H17 and H18 subtypes with few sequences were not considered in our analysis.

To validate the reasonability of our datasets, we constructed the approximately-maximum-likelihood phylogenetic trees of each protein with FastTree 2.1 (http://www.microbesonline.org/fasttree/). For each of the internal proteins, three sub-clades could be achieved: avian clade, human H1N1 clade, and human H3N2 clade (Figure S3). For the HA or NA protein, two groups were achieved in either avian or human dataset (Figures S4 and S5).

Sequences of each internal protein were aligned by MAFFT version 7^45^. Because of low sequence similarities between subtypes of HA(NA), a structure based sequence alignment should be constructed, in which case sequences of HA (NA) were added into using MAFFT with ‘–add’ parameter. Structure based sequence alignment for HA described in literature^46^ was used. As for NA, crystal structures of N1 to N9 were downloaded from PDB database^47^ and aligned with structure alignment tool DeepAlign^48^.

### Identification of specific sites

Given a column of two aligned sequence sets (set A and set B), the frequencies of residues of the column in each set were counted. The dScore was defined to assess the difference of a certain site between two sets with the following formula subsequently:

1 $${\rm{dscore}}\,({\rm{c}})=1-\,\sum _{r\in R}\,{\rm{\min }}({f}_{A}(r,c),{f}_{B}(r,c))$$

*r* is an arbitrary residue in the standard amino acids set R. f_A_(r,c) is denoted as the frequency of residue r in column c of in set A while f_B_(r,c) is the frequency of residue r in column c of in set B. dScore(c) ranges from 0 to 1.The more the dScore(c) approximates to 1, the greater different the site c between two sets is.

To balance the sequences between different lineages or subtypes, we used a bootstrap sample method. First of all, 500 sequences of each lineage or subtype were sampled with replication and performed one calculation of dScore with equation (1). This procedure was repeated 1000 times and an average dScore was obtained. Finally, sites with average dScore more than 0.90 were selected (Figure S1).

### Homology modeling

We predicted the protein structures using the side-chain modeling tool CISRR^49^. Crystal structures with high resolutions (<3.0Å) were selected as templates in priority (Table S5).

### Identification of H-Bonds

H-bonds were identified using the simple geometric criteria of Baker and Hubbard^11^.The distance between donor atom and acceptor atom^50^ is less than 3.5 Å and the angle between the donor antecedent, donor and acceptor 90–180°. Main-chain and main-chain H-bonds were not considered in our analysis.

### Calculation of Relative Solvent Accessibility

Relative solvent accessibility (RSA) of a residue was calculated using the program NAccess (unpublished, S. Hubbard and J. Thornton 1992–6, http://www.wolf.bms.umist.ac.uk/naccess/) and ACCpro^51^. A site was regarded as exposed if its RSA was above 25%^52^.

## Electronic supplementary material

Supplementary Materials
