# Supplementary material for: Hydrogen Bond Variations of Influenza A Viruses During Adaptation in Human
Source: Sci Rep. 2017 Oct 30;7:14295. doi: 10.1038/s41598-017-14533-3 (PMC5662722; doi:10.1038/s41598-017-14533-3)
Supplement: Supplementary file 1 — Supplementary Materials [file 41598_2017_14533_MOESM1_ESM.pdf]

Hydrogen Bond Variations of Influenza A Viruses During Adaptation in Human

Jiejian Luo[1,2†], Lizong Deng[3†], Xiao Ding[3], Lijun Quan[3], Aiping Wu[3*], Taijiao Jiang[1,3*]

[1] Key Laboratory of Protein & Peptide Pharmaceuticals, National Laboratory of Biomacromolecules, Institute of Biophysics, Chinese Academy of Sciences, Beijing, China;
[2] University of the Chinese Academy of Sciences, Beijing, China;
[3] Center for Systems Medicine, Institute of Basic Medical Sciences, Chinese Academy of Medical Sciences & Peking Union Medical College, Beijing 100005; Suzhou Institute of Systems Medicine, Suzhou, Jiangsu 215123, China

† These authors contributed equally to this work.
* Correspondence: Taijiao Jiang, taijiao@ibms.pumc.edu.cn or Aiping Wu, wap@ism.cams.cn

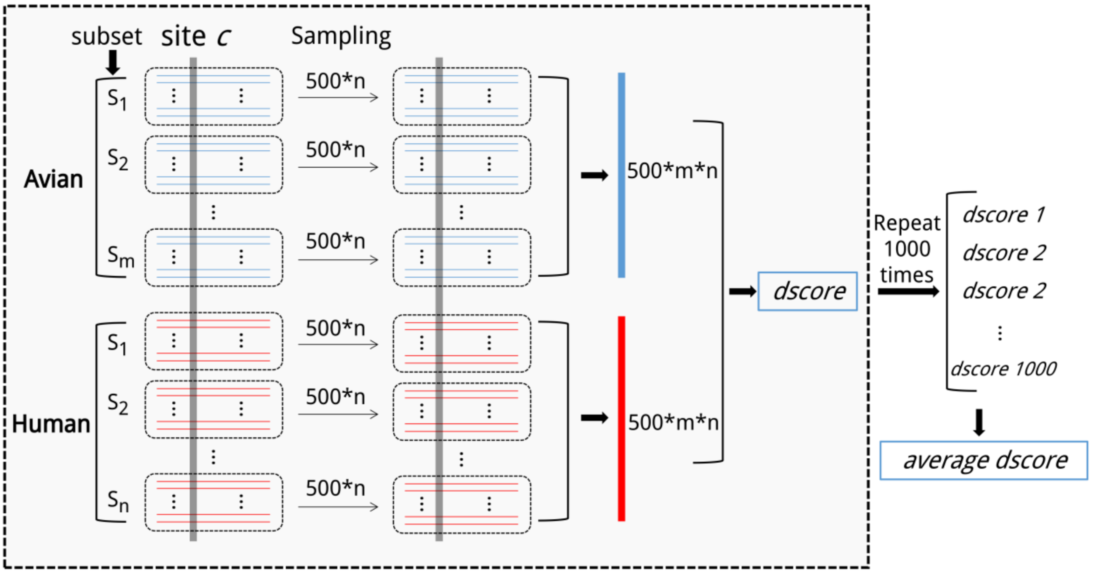

Figure. S1. The schematic diagram of balancing the sequences between different lineages or subtypes.

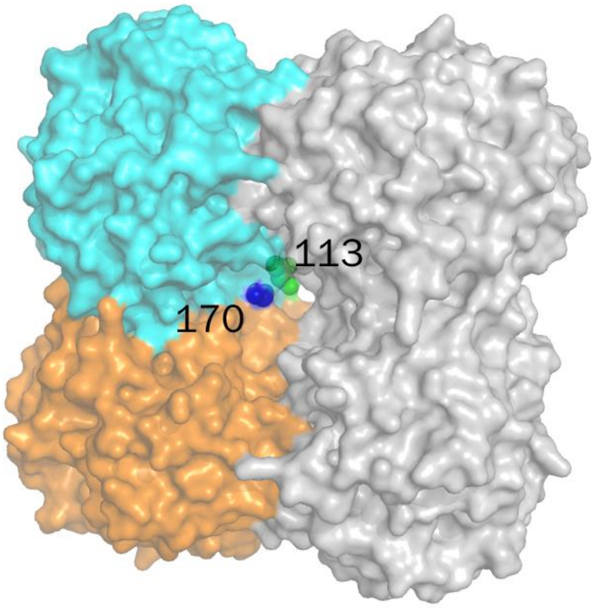

Figure. S2. Three-dimensional structure of influenza NA protein (PDB: 3TIA). The 3D structure of NA protein was depicted in surface mode. Two monomers were shown in cyan and orange, respectively. The site 170 and 113 that was located on the interface of subunits was labeled as blue and green sphere, respectively.

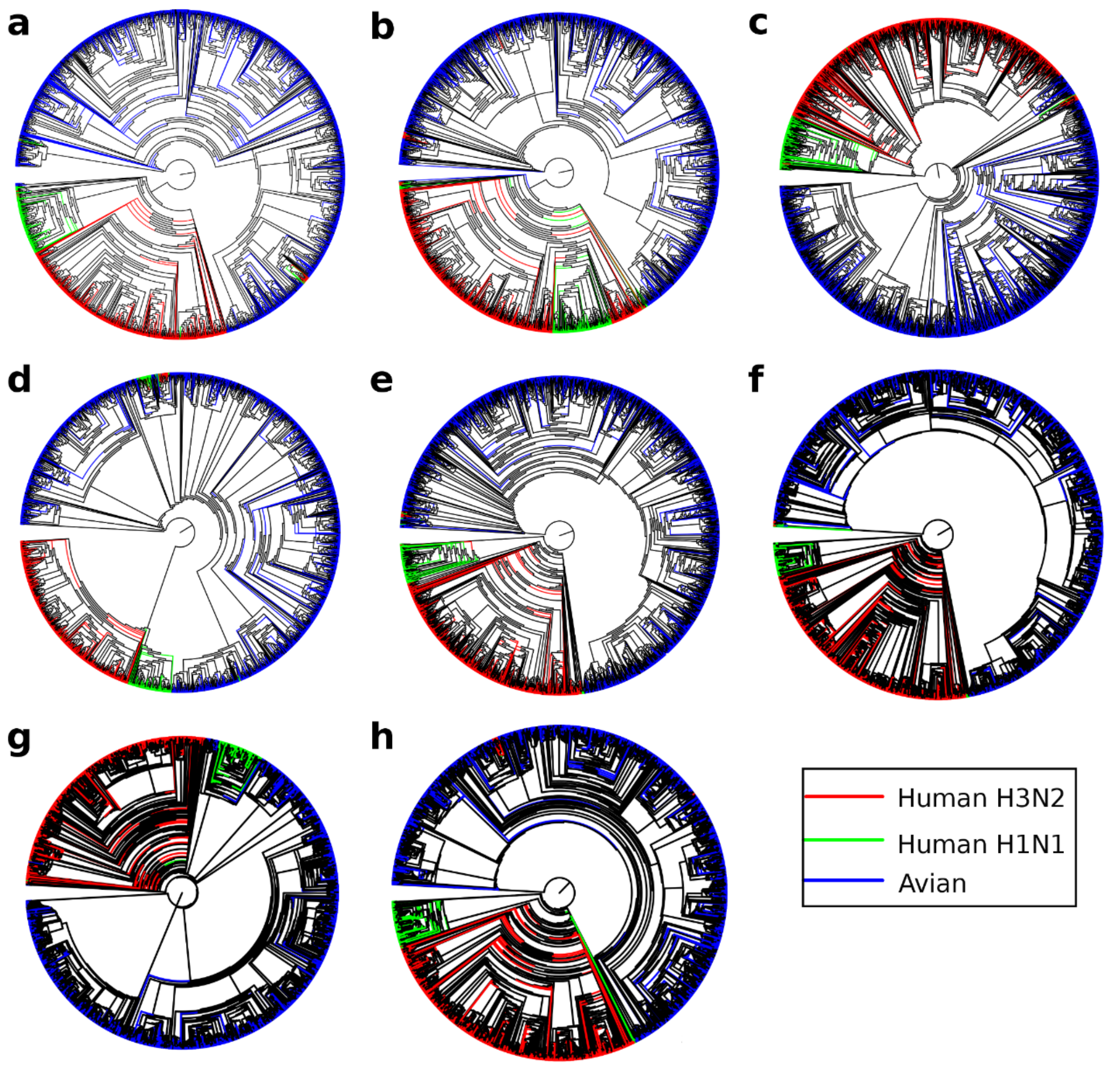

Figure S3. Approximately-maximum-likelihood phylogenetic trees of eight internal proteins. a) M1; b) M2; c) NS1; d) NS2; e) NP; f) PA; g) PB1; h) PB2. All trees were shown in polar tree layout with FigTree 1.42. Human H3N2, H1N1, and avian strains were colored as red, green, and blue branches, respectively.

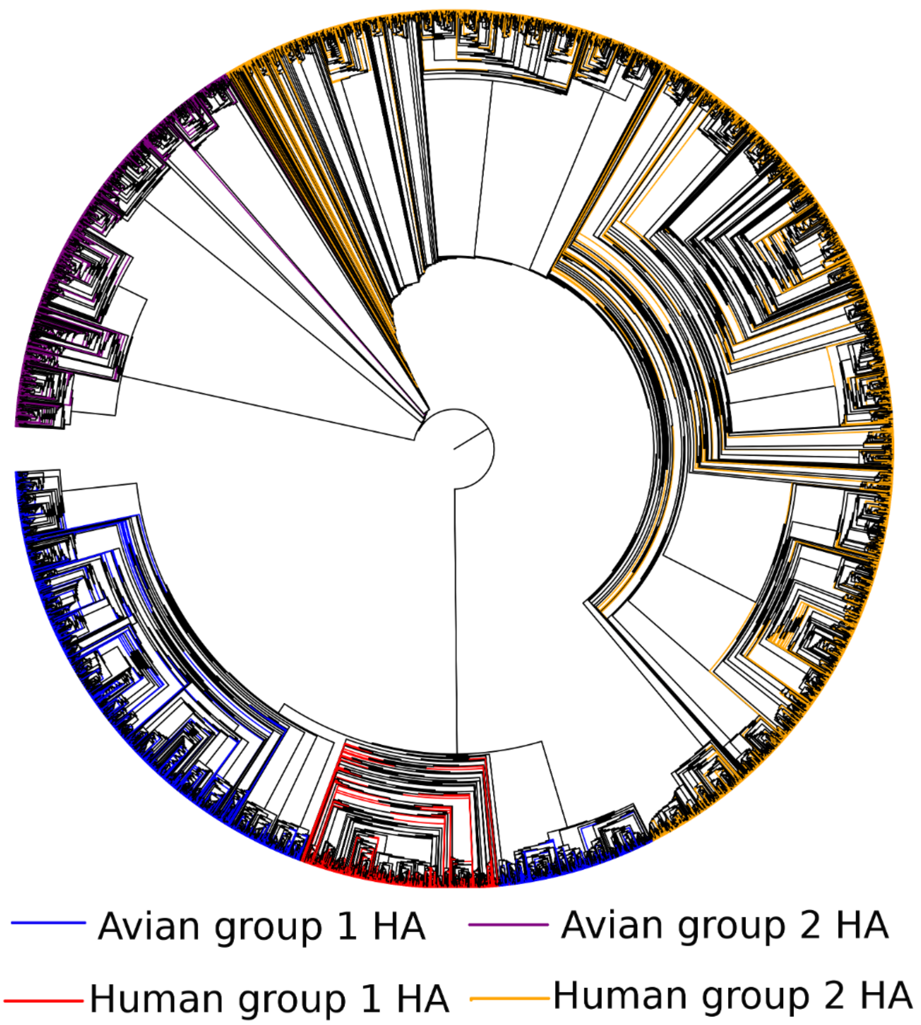

Figure S4. Approximately-maximum-likelihood phylogenetic tree of HA. The tree was shown in polar tree layout with FigTree 1.42. The groups of avian and human HA were colored as blue, purple, red, and orange branches, respectively. Subtypes in each group were listed as follow: Avian group 1 HA (H1, H2, H5, and H6); Avian group 2 HA (H3, H4, H7, H10); Human group 1 HA (H1), and Human group 2 HA (H3).

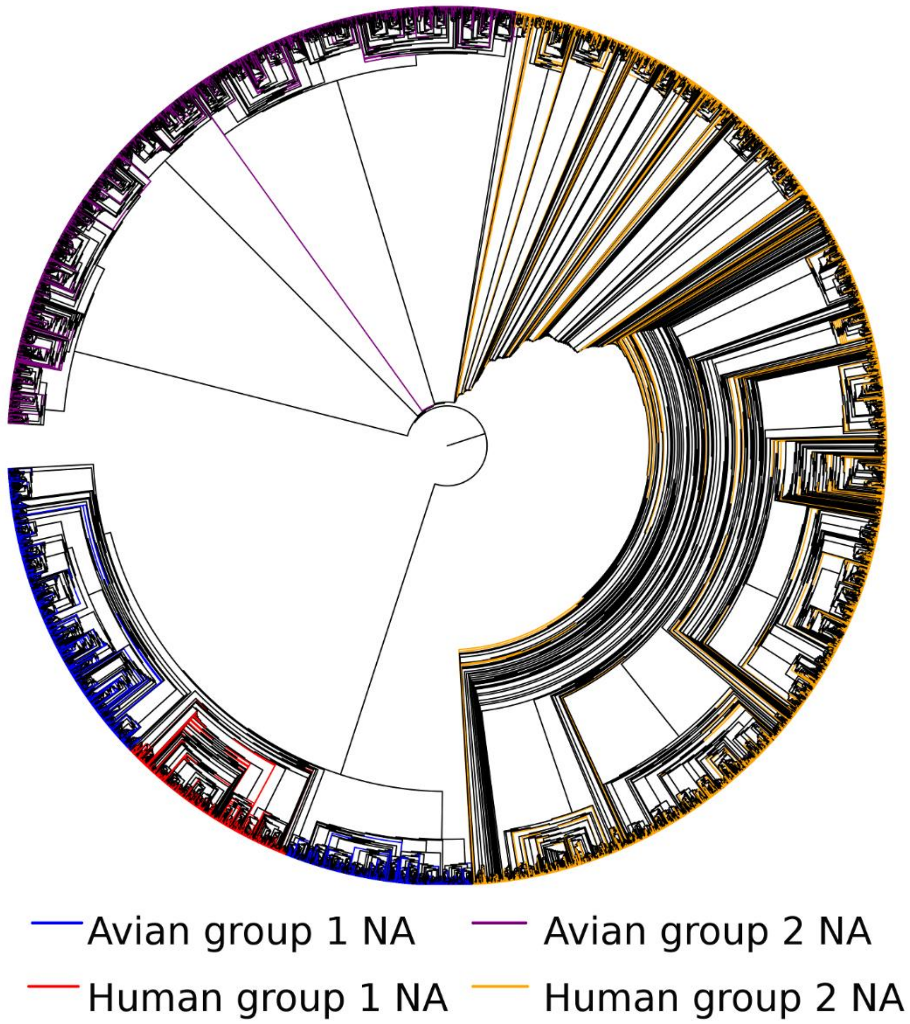

Figure S5. Approximately-maximum-likelihood phylogenetic tree of NA. The tree was shown in polar tree layout with FigTree 1.42. The groups of avian and human NA were colored as blue, purple, red, and orange branches, respectively. Subtypes in each group were listed as follow: Avian group 1 NA (N1, N4, N5, N8); Avian group 2 NA (N2, N3, N6, N7, N9); Human group 1 NA (N1), and Human group 2 NA (N2).

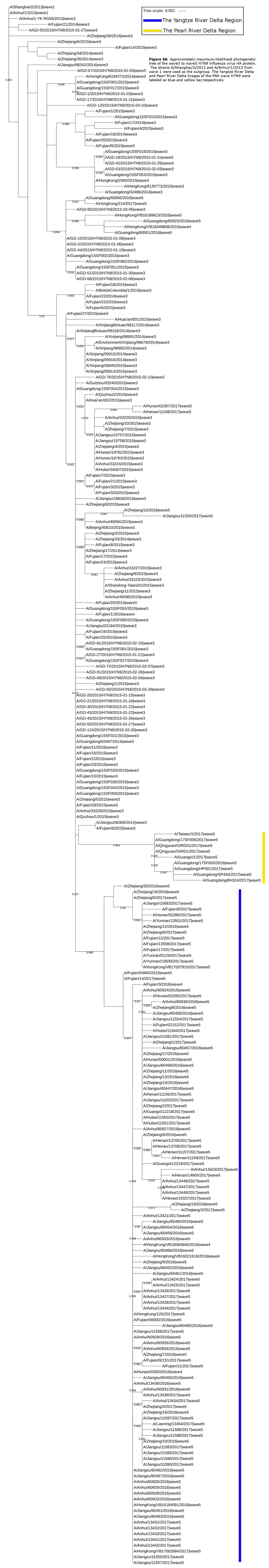

Figure S6. Approximately-maximum-likelihood phylogenetic tree of the wave3 to wave5 H7N9 influenza virus HA protein. The strains A/Shanghai/2/2013 and A/Anhui/1/2013 from wave 1 were used as the outgroup. The Yangtze River Delta and Pearl River Delta linages of the fifth wave H7N9 were labeled as blue and yellow bar,respectively.

Table S1. Sequences of HA groups used in our analysis.

Group	Subgroup	Human		Avian	
		Subtype	Number	Subtype	Number
group 1	g1.1	H1	1560	H6	1080
				H5	3617
				H2	295
				H1	139
	g1.2	-	-	H16	96
				H13	148
				H11	498
	g1.3	-	-	H9	1951
				H12	159
				H8	116
group 2	g2.1	-	-	H7	1153
				H15	7
				H10	489
	g2.2	H3	10837	H3	819
				H4	820
				H14	16

Table S2. H-bond variations at group specific sites of HA and NA. "-" means that the site is missing in predicted structures. The star "*" after the site number means a deletion after this site. The format of H-bonds is "HB(donor residue, acceptor residue, donor atom–H...acceptor atom)".

group 1 HA:

Mutation	H1		H2		H5		H6	
	loss	formation	loss	formation	loss	formation	loss	formation
D77E	HB(149R, 77D, Nε-H...Oδ1);<br>HB(149R, 77D, Nη2-H...Oδ2)	HB(141Y, 77E, Oη-H...Oε1)	HB(149R, 77D, Nε-H...Oδ1);<br>HB(149R, 77D, Nε-H...Oδ2);<br>HB(149R, 77D, Nη2-H...Oδ2)	HB(149R, 77E, Nη1-H...Oε2)	HB(149R, 77D, Nη1-H...Oδ1);<br>HB(149R, 77D, Nη1-H...Oδ2)	HB(141Y, 77E, Oη-H...Oε1)	HB(149R, 77D, Nε-H...Oδ1);<br>HB(149R, 77D, Nη2-H...Oδ2)	HB(149R, 77E, Nη1-H...Oε2)
K156G	HB(156K, 192Q, Nζ-H...O)				HB(156K, 192T, Nζ-H...O);<br>HB(156K, 196Q, Nζ-H...Oε1)		HB(156K, 192N, Nζ-H...O)	
K156E	HB(156K, 192Q, Nζ-H...O)	HB(196Q, 156E, Nε2-H...Oε2);<br>HB(196Q, 156E, N-H...Oε1)			HB(156K, 192T, Nζ-H...O);<br>HB(156K, 196Q, Nζ-H...Oε1)	HB(160S, 156E, N-H...Oε1);<br>HB(196Q, 156E, N-H...Oε2)	HB(156K, 192N, Nζ-H...O)	
E190D			HB(186N, 190E, N-H...Oε1)		HB(186N, 190E, Nδ2-H...Oε1);<br>HB(186N, 190E, N-H...Oε2)	HB(186N, 190D, Nδ2-H...Oδ2)		
E190N			HB(186N, 190E, N-H...Oε1)	HB(190N, 187D, Nδ2-H...Oδ2)	HB(186N, 190E, Nδ2-H...Oε1);<br>HB(186N, 190E, N-H...Oε2)			
G205V								
G225D								
K310R			HB(310K, 419D, Nζ-H...Oδ1)	HB(310R, 419D, Nη2-H...Oδ1)	HB(310K, 419D, Nζ-H...Oδ1)	HB(310R, 415D, Nη1-H...Oδ1);<br>HB(310R, 419D, Nη2-H...Oδ1)		HB(310R, 419D, Nη2-H...Oδ1)
A317V								
N401K	HB(402L, 401N, N-H...Oδ1)		HB(402L, 401N, N-H...Oδ1)		HB(402L, 401N, N-H...Oδ1)	HB(404R, 401K, Nε-H...O)		

Table S2 Cont.

group 2 HA:

Mutation	H3		H4		H7		H10	
	loss	formation	loss	formation	loss	formation	loss	formation
E190D	HB(186S, 190E, Oγ-H...Oε1); HB(186S, 190E, N-H...Oε1)	HB(186S, 190D, Oγ-H...Oδ2)	HB(186S, 190E, N-H...Oε1)		HB(186G, 190E, N-H...Oε1)		HB(186S, 190E, Oγ-H...Oε1)	HB(186S, 190D, Oγ-H...Oδ2)
G225D								HB(137K, 225D, Nζ-H...Oδ2)
G225N								
Q226I	HB(226Q, 98Y, Nε2-H...Oη)				HB(136T, 226Q, Oγ1-H...Oε1); HB(226Q, 136T, Nε2-H...Oγ1)			
G228S		HB(186S, 228S, N-H...Oγ); HB(226Q, 228S, Nε2-H...Oγ); HB(228S, 190E, Oγ-H...Oε1); HB(228S, 190E, Oγ-H...Oε2)		HB(98Y, 228S, Oη-H...Oγ); HB(228S, 226Q, Oγ-H...Oε1)		HB(98Y, 228S, Oη-H...Oγ); HB(186G, 228S, N-H...Oγ); HB(228S, 190E, Oγ-H...Oε1)		HB(98Y, 228S, Oη-H...Oγ)
L331I	HB(331L, 438D, N-H...Oδ1)				-	-		
E386G								
E479G			HB(482R, 479E, Nε-H...Oε1); HB(482R, 479E, Nη2-H...Oε1); HB(483N, 479E, Nδ2-H...Oε1); HB(483N, 479E, Nδ2-H...Oε2)		HB(482R, 479E, Nη2-H...Oε2)			

Table S2 Cont.

group 1 NA:

Mutation	N1		N4		N5		N8	
	loss	formation	loss	formation	loss	formation	loss	formation
S101T	HB(101S, 445S, Oγ-H...Oγ);<br>HB(102K, 101S, N-H...Oγ)	HB(102K, 101T, N-H...Oγ1)		HB(102K, 101T, N-H...Oγ1)				
D213G	HB(214T, 213D, N-H...Oδ1);<br>HB(262V, 213D, N-H...Oδ2)		HB(214T, 213D, N-H...Oδ1);<br>HB(262I, 213D, N-H...Oδ2)		HB(214V, 213D, N-H...Oδ1);<br>HB(262V, 213D, N-H...Oδ2)		HB(214V, 213D, N-H...Oδ1);<br>HB(262I, 213D, N-H...Oδ2)	
D213E	HB(214T, 213D, N-H...Oδ1);<br>HB(262V, 213D, N-H...Oδ2)	HB(214T, 213E, N-H...Oε1);<br>HB(215I, 213E, N-H...Oε2)	HB(214T, 213D, N-H...Oδ1);<br>HB(262I, 213D, N-H...Oδ2)	HB(214T, 213E, N-H...Oε1)	HB(214V, 213D, N-H...Oδ1);<br>HB(262V, 213D, N-H...Oδ2)	HB(214V, 213E, N-H...Oε1)	HB(214V, 213D, N-H...Oδ1);<br>HB(262I, 213D, N-H...Oδ2)	HB(262I, 213E, N-H...Oε1); HB(262I,<br>213E, N-H...Oε2)
Q249A	HB(249Q, 250A, Nε2-H...O);<br>HB(249Q, 252Y, Nε2-H...Oη);<br>HB(249Q, 270A, Nε2-H...O);<br>HB(249Q, 273Y, Nε2-H...O);<br>HB(272N, 249Q, N-H...Oε1)		HB(249Q, 273F, Nε2-H...O);<br>HB(250A, 249Q, N-H...Oε1)		HB(249Q, 250A, Nε2-H...O);<br>HB(249Q, 252Y, Nε2-H...Oη);<br>HB(249Q, 270F, Nε2-H...O);<br>HB(249Q, 273G, Nε2-H...O);<br>HB(272G, 249Q, N-H...Oε1)		HB(249Q, 250A, Nε2-H...O);<br>HB(249Q, 252Y, Nε2-H...Oη);<br>HB(249Q, 270F, Nε2-H...O);<br>HB(249Q, 273G, Nε2-H...O);<br>HB(272G, 249Q, N-H...Oε1)	
T334E		HB(387T, 334E, Oγ1-H...Oε2)						
T334K								
Y347N				HB(346R, 347N, Nη1-H...Oδ1)	HB(371R, 347Y, Nη2-H...Oη)	HB(292R, 347N, Nη2-H...Oδ1);<br>HB(348G, 347N, N-H...Oδ1)	HB(371R, 347Y, Nη1-H...Oη)	
Y347D		HB(292R, 347D, Nη2-H...Oδ1);<br>HB(348G, 347D, N-H...Oδ1)		HB(346R, 347D, Nη1-H...Oδ1)	HB(371R, 347Y, Nη2-H...Oη)	HB(292R, 347D, Nη2-H...Oδ1);<br>HB(348G, 347D, N-H...Oδ1)	HB(371R, 347Y, Nη1-H...Oη)	HB(292R, 347D, Nη2-H...Oδ1);<br>HB(348G, 347D, N-H...Oδ1)
F354Y		HB(354Y, 410F, Oη-H...O);<br>HB(354Y, 421C, Oη-H...O);<br>HB(410F, 354Y, N-H...Oη)		HB(354Y, 410F, Oη-H...O);<br>HB(354Y, 421C, Oη-H...O);<br>HB(410F, 354Y, N-H...Oη);<br>HB(421C, 354Y, N-H...Oη)		HB(354Y, 410F, Oη-H...O);<br>HB(354Y, 421C, Oη-H...O);<br>HB(410F, 354Y, N-H...Oη);<br>HB(421C, 354Y, N-H...Oη)		HB(354Y, 409S, Oη-H...Oγ);<br>HB(354Y, 410F, Oη-H...O);<br>HB(354Y, 421C, Oη-H...O);<br>HB(410F, 354Y, N-H...Oη)
S370L/I	HB(370S, 372S, Oγ-H...Oγ);<br>HB(371R, 370S, N-H...Oγ)		HB(432K, 370S, Nζ-H...Oγ)		HB(370S, 367S, Oγ-H...Oγ);<br>HB(370S, 372S, Oγ-H...Oγ)		HB(370S, 367S, Oγ-H...Oγ)	
S372K	HB(370S, 372S, Oγ-H...Oγ)		HB(400N, 372S, Nδ2-H...Oγ);<br>HB(400N, 372S, Nδ2-H...O)		HB(370S, 372S, Oγ-H...Oγ);<br>HB(372S, 400N, Oγ-H...Oδ1)		HB(372S, 400N, Oγ-H...Oδ1);<br>HB(372S, 400N, Oγ-H...O)	
I427V								

Table S2 Cont.

group 2 NA:

Mutation	N2		N3		N6		N7		N9	
	loss	formation	loss	formation	loss	formation	loss	formation	loss	formation
I56T	-	-	-	-	-	-	-	-	-	-
G147N/D				HB(430R, 147N, Nη1-H...Oδ1)					HB(150H, 147G, Nδ1-H...O)	
I149V										
N400R	HB(372S, 400N, Oγ-H...Oδ1)		HB(400N, 372S, Nδ2-H...Oγ); HB(400N, 372S, Nδ2-H...O)		HB(367S, 400N, N-H...Oδ1); HB(372S, 400N, Oγ-H...Oδ1)		HB(372S, 400N, Oγ-H...Oδ1)		HB(367S, 400N, N-H...Oδ1); HB(372S, 400N, Oγ-H...Oδ1)	
W403R		HB(403R, 433E, Nη1-H...Oε1); HB(403R, 433E, Nη2-H...Oε1)		HB(403R, 431*K, Nη1-H...O)		HB(403R, 433E, Nη1-H...Oε2)	HB(403W, 433E, Nε1-H...Oε2)	HB(403R, 429G, Nη2-H...O); HB(403R, 433E, Nη2-H...Oε1)		HB(403R, 429G, Nη2-H...O); HB(403R, 433E, Nη2-H...Oε1)
P431K										

Table S3. Protein sequences of IAVs used in our datasets. For human IAVs, pandemic isolates were not considered. For avian IAVs, suspicious strains such as avian-host H1N1, H2N2 and H3N2 were excluded. The human H2N2 subtype without enough sequences was discarded in the following analysis.

Protein	Human			Avian
	H1N1	H3N2	H2N2	HxNx
HA	1560	10837	41	11403
M1	165	537	16	1635
M2	253	919	14	2184
NA	1393	9512	50	9201
NP	294	1442	46	4482
NS1	441	2478	34	4479
NS2	171	534	16	1939
PA	411	2643	36	7733
PB1	512	2737	36	6973
PB2	534	3167	40	7574

Table S4. Sequences of NA groups used in our analysis.

Group	Human		Avian	
	Subtype	Number	Subtype	Number
group 1	N1	1393	N1	2321
			N4	141
			N5	221
			N8	1054
group 2	N2	9512	N2	2546
			N3	714
			N6	1239
			N7	459
			N9	506

Table S5. Templates used for homology modeling.

Protein	PDB ID	Resolution(Å)	Chains For Modeling
H1	4GXX	1.8	AB
H2	3KU3	1.6	AB
H3	4O5N	1.75	AB
H5	4JUM	2	AB
H6	5BR0	2.39	AB
H7	4LN6	2.12	AB
H9	1JSD	1.8	AB
H10	4CYV	2.3	AB
H13	4KPQ	2.5	AB
H16	4FIU	2	A
N1	3BEQ	1.64	A
N2	3TIA	1.8	A
N3	4HZV	1.8	A
N4	2HTV	2.8	A
N5	3SAL	1.5	A
N6	5HUM	1.6	A
N7	4QN3	2.09	A
N8	4WA3	1.8	A
N9	4MWJ	1.8	A
NP	4IRY	2.8	A
NS1	4OPA	2.7	A
NS2	1PD3	2.6	A
M1	2Z16	2.02	A
M2	2L0J	Solid-State-NMR	ABCD
polymerase	4WSB	2.65	ABC

Table S6. Representative strains used in Figure. 3c.

Subtype	Strain Name	Year
H5N1	A/Hong Kong/481/97	1997
H5N1	A/Hong Kong/485/97	1997
H5N1	A/Hong Kong/532/1997	1997
H5N1	A/Hong Kong/542/97	1997
H5N1	A/Hong Kong/156/97	1997
H5N1	A/Beijing/01/2003	2003
H5N1	A/Vietnam/UT3028/2003	2003
H5N1	A/Thailand/1(KAN-1)/2004	2004
H5N1	A/Viet Nam/1203/2004	2004
H5N1	A/Anhui/1/2005	2005
H5N1	A/Fujian/1/2005	2005
H5N1	A/Indonesia/5/2005	2005
H5N1	A/Jiangxi/1/2005	2005
H5N1	A/Thailand/676/2005	2005
H5N1	A/Vietnam/UT30850/2005	2005
H5N1	A/Hunan/1/2006	2006
H5N1	A/Indonesia/292H/2006	2006
H5N1	A/Shanghai/1/2006	2006
H5N1	A/Thailand/NBL1/2006	2006
H5N1	A/Xinjiang/1/2006	2006
H5N1	A/Zhejiang/1/2006	2006
H5N1	A/Anhui/1/2007	2007
H5N1	A/Fujian/1/2007	2007
H5N1	A/Jiangsu/1/2007	2007
H5N1	A/Vietnam/UT31239/2007	2007
H5N1	A/Guangdong/1/2008	2008
H5N1	A/Guangxi/1/2008	2008
H5N1	A/Hunan/1/2008	2008
H5N1	A/Indonesia/7261/2008	2008
H5N1	A/Vietnam/UT31412II/2008	2008
H5N1	A/Egypt/N11981/2009	2009
H5N1	A/Guizhou/1/2009	2009
H5N1	A/Hunan/1/2009	2009
H5N1	A/Xinjiang/1/2009	2009
H5N1	A/Egypt/N01644/2010	2010
H5N1	A/Hubei/1/2010	2010
H5N1	A/Indonesia/NIHRD10364/2010	2010
H5N1	A/Bangladesh/3233/2011	2011
H5N1	A/Egypt/N0423/2011	2011
H5N1	A/Indonesia/NIHRD11046/2011	2011
H5N1	A/Viet Nam/CM32/2011	2011
H5N1	A/Cambodia/W0112303/2012	2012
H5N1	A/Hong Kong/5923/2012	2012
H5N1	A/Vietnam/VP12-3/2012	2012
H5N1	A/Cambodia/X0207301/2013	2013
H5N1	A/Vietnam/VP39/2013	2013
H5N1	A/Egypt/MOH-NRC-7271/2014	2014
H5N1	A/Vietnam/14011801/2014	2014
H5N1	A/Egypt/682/2015	2015

Table S7. Representative strains used in Figure. 3d and Figure. 4a.

Subtype	Strain Name	Year	Month	Wave
H7N9	A/Anhui/1/2013	2013	3	wave 1
H7N9	A/Jiangsu/01/2013	2013	3	wave 1
H7N9	A/Zhejiang/1/2013	2013	3	wave 1
H7N9	A/Fujian/1/2013	2013	4	wave 1
H7N9	A/Henan/01/2013	2013	4	wave 1
H7N9	A/Hunan/01/2013	2013	4	wave 1
H7N9	A/Shandong/01/2013	2013	4	wave 1
H7N9	A/Taiwan/S02076/2013	2013	4	wave 1
H7N9	A/Guangdong/1/2013	2013	8	wave 1
H7N9	A/Fujian/3/2014	2014	1	wave 2
H7N9	A/Guangdong/0010/2014	2014	1	wave 2
H7N9	A/Shanghai/01/2014	2014	1	wave 2
H7N9	A/Zhejiang/1/2014	2014	1	wave 2
H7N9	A/Hong Kong/3263/2014	2014	2	wave 2
H7N9	A/Taiwan/1/2014	2014	4	wave 2
H7N9	A/Xinjiang/73030/2014	2014	7	wave 2
H7N9	A/Zhejiang/33/2014	2014	11	wave 3
H7N9	A/Fujian/21/2014	2014	12	wave 3
H7N9	A/Guangdong/15SF017/2015	2015	1	wave 3
H7N9	A/Hong Kong/2550/2015	2015	1	wave 3
H7N9	A/Zhejiang/4/2015	2015	1	wave 3
H7N9	A/Anhui/33227/2015	2015	2	wave 3
H7N9	A/Huai'an/002/2015	2015	2	wave 3
H7N9	A/Hunan/19762/2015	2015	2	wave 3
H7N9	A/Fujian/1/2016	2016	1	wave 4
H7N9	A/Fujian/3/2016	2016	1	wave 4
H7N9	A/Hunan/02650/2016	2016	1	wave 4
H7N9	A/Hong Kong/VB16021618/2016	2016	2	wave 4
H7N9	A/Hong Kong/VB16049808/2016	2016	3	wave 4
H7N9	A/Hong Kong/VB16064646/2016	2016	4	wave 4
H7N9	A/Zhejiang/6/2016	2016	10	wave 5
H7N9	A/Guangdong/60060/2016	2016	12	wave 5
H7N9	A/Guangdong/60923/2016	2016	12	wave 5
H7N9	A/Hunan/00001/2016	2016	12	wave 5
H7N9	A/Jiangsu/60452/2016	2016	12	wave 5
H7N9	A/Fujian/02151/2017	2017	1	wave 5
H7N9	A/Fujian/02152/2017	2017	1	wave 5
H7N9	A/Hong Kong/214/2017	2017	1	wave 5
H7N9	A/Hunan/02285/2017	2017	1	wave 5
H7N9	A/Hunan/02286/2017	2017	1	wave 5
H7N9	A/Qingyuan/GIRD1/2017	2017	1	wave 5
H7N9	A/Zhejiang/2/2017	2017	1	wave 5
H7N9	A/Zhejiang/4/2017	2017	1	wave 5
H7N9	A/Zhejiang/5/2017	2017	1	wave 5
H7N9	A/Taiwan/1/2017	2017	2	wave 5

Table S8. Differential sites and H-bond variations between the PRD and YRD lineages in the fifth H7N9 wave. The H-bond variations were assessed with the AnH1 as reference. The status of H-bond variations was shown in different color. Gray: no changes; Red: H-bond formation; Light blue: H-bond loss; Green: H-bond formation & H-bond loss. The details of H-bonds were not shown.

Protein	Site†	AnH1#	PRD‡	YRD*
H7	48	I	T(9/9)	I(114/114)
	122	A	P(9/9)	T(99/114)
	140	R	R(9/9)	K(114/114)
	173	K	E(9/9)	K(114/114)
	226	L	Q(9/9)	L(113/114)
	**324-330**	PEIPKGRG	PEVPKRKRTARG(9/9)	PEIPKGRG(114/114)
	393	E	K(9/9)	E(114/114)
	429	V	V(9/9)	I(114/114)
M1	**168**	T	S(8/9)	T(114/114)
M2	**80**	Q	R(8/9)	Q(114/114)
N9	**21**	A	T(8/9)	A(114/114)
	**39**	P	S(8/9)	P(114/114)
	**44**	H	R(8/9)	H(113/114)
	170	Y	Y(8/9)	H(99/114)
	205	V	I(8/9)	V(114/114)
	283	E	K(8/9)	E(113/114)
	292	R	K(8/9)	R(111/114)
	430	R	K(8/9)	R(112/114)
PA	262	R	K(8/9)	R(106/114)

†: Sites in bold were missing in the predicted homology structures. The assessment of H-bond variations at these sites was skipped.

#: The residues of AnH1 (A/Anhui/1/2013).

‡: Dominant residues of the fifth wave H7N9 strains of the Pearl River Delta Linages(PRD).

*: Dominant residues of the fifth wave H7N9 strains of the Yangtze River Delta Linages(YRD).
